# Impacts of mining disasters on the ambulatory care of the Brazilian national health system: the cases of Mariana and Brumadinho/Brazil

**DOI:** 10.1186/s12913-023-10385-y

**Published:** 2024-03-05

**Authors:** Emerson Pessoa Vidal, Rita de Cássia Costa da Silva, Paola Zucchi

**Affiliations:** 1 Graduate Program in Translational Medicine, Department of Medicine, Federal University of Sao Paulo (Unifesp), São Paulo, Brazil; 2 Department of Medicine - Discipline of Economics and Management in Health, Federal University of São Paulo (Unifesp), São Paulo, Brazil

**Keywords:** Man-made Disasters, Disaster Management, Impact of Disasters, Ambulatory care, Health expenditures

## Abstract

**Background:**

Disasters are events that bring with them effects that contribute to the disruption of the normality of a population and thus highlight the vulnerabilities of the health system. In Mariana and Brumadinho, the collapse of the dam of ore tailings brought with it several impacts that were felt in the short term and will be felt in the medium and long term. And that by being intensely intertwined with issues of economic and productive nature, has as its meaning an uninterrupted result of its activities.

**Methods:**

Through the DATASUS database, two specific variables were chosen to perform the analysis: the approved amount and the approved value. For this research, a methodological device, the segmented regression line, was used to observe the influences that the disasters that occurred in Mariana and Brumadinho had on the ambulatory health systems.

**Results:**

The results of the segmented regression line show that, with Mariana, the amount approved continued to grow throughout the period, which shows that there was no change because of the disaster. There was a reduction in spending. In Brumadinho, regarding the amount approved, there was an upward trend in the disaster’s month, which did not change immediately afterwards, and regarding expenditure, the growth pattern was maintained in all three periods. Corroborating this data, the relative and absolute base elements show an increase in the amount approved and in the number of services provided at various posts compared with Minas Gerais.

**Conclusions:**

Based on the findings, it was possible to understand that although disasters exert an influence that may have some effect on the health system, the lack of significance sometimes cannot be interpreted as a lack of impact on the disaster. The segmented regression line outlines some effects that are not conclusive but indicative of a numerical interpretation and a trend interpretation.

## Introduction

Disasters are events that disrupt the normality of a population, and when they occur, they can bring to light the vulnerabilities of the health system to respond in a timely manner to people’s health needs.

In fact, a disaster brings with it effects that can contribute to the fact that integral health care is not offered in its fullness, thus being a threat not only to the material conditions of the population but also to the most basic rights.

In the case of mining disasters such as those that occurred in Mariana and Brumadinho, the collapse of the tailings dam brought with it, in addition to flooding and contamination, several short, medium, and long-term impacts that are still being felt by the population.

For Rocha and Londe, disasters that involve technological threats are related to production cycles that stimulate the economy, and in the case of mining, it involves the production process, extraction and disposal or disposal of waste, and the transport of products from the entry into another production chain [1]. Since it involves contaminants in the soil, water, or air, which can remain for long periods of time, with rapid or delayed effects, technological disasters can cause threats that require special attention from the point of view of public health. In addition to generating direct impacts (injuries, trauma and death), the number of victims can exceed the response capacity of the local emergency system. Moreover, in the medium and long term, new health needs may arise when the necessary measures to curb secondary risks are not taken immediately [1].

Between 2002 and 2011, approximately 7,000 disasters were reported worldwide, causing more than 1.2 million lives lost and more than 2.5 million people to be homeless, economically impacted, or otherwise harmed. The consequences on the health of affected communities can be complex and directly impact them, in addition to changing the morbidity and mortality profile and impacting the health system [2].

For Cerutti and Oliveira, interruption in the functioning of health services can be the difference between life and death. For services to be offered in situations that involve major accidents, the institution must have the structure and trained personnel to continue providing care even under such circumstances [3]. In Brazil, the Unified Health System is responsible for the immediate care of disaster victims, which reinforces the importance of these observations so that citizens have access to and assurance of integral care in the public health system.

On November 5, 2015, in the municipality of Mariana in Minas Gerais, Brazil, the Fundão dam, operated by the Samarco mining company, burst, releasing approximately 50 million m3 of iron mining tailings and killing 19 people. It was one of the biggest environmental disasters in Brazil [2]. Three years later, on January 25, 2019, another disaster occurred at the Córrego do Feijão Mine in Brumadinho, also in Minas Gerais, subjecting the community to a tragedy with incalculable damage [4]. Data released by the Association of Families of Victims and People Affected by the Córrego do Feijão Dam (AVABRUM) registered 272 deaths, with three victims still missing [5, 6].

In the Mariana tragedy, besides destroying villages like Bento Rodrigues and Paracatu de Baixo, the mud covered 663 km along the Gualaxo do Norte, Carmo, and Doce rivers, reaching the mouth of the latter. The mud affected the ecosystem in regions where marine species reproduce. Thirty-five municipalities in Minas Gerais and four in Espírito Santo were affected, with approximately 1.2 million people affected by the lack of water and who feared contamination of the Doce River water, which was returned to supply and consume the water. In Brumadinho, the largest work accident with loss of human life, the disaster caused 272 deaths, making the country the highest ranking in deaths directly related to the mining disaster [7].

Iron ore extraction does not only produce billions of dollars and “progress”; it is also full of threats, deaths, and socio-environmental destruction. Employees die and get sick; large areas are deforested; trucks and trains run over individuals and animals; the processing plants generate atmospheric pollution; aquifers formed in ferriferous regions are contaminated and destroyed; in times of water problems, the water consumed is enormous, including by the pipelines; the sum of the tailings is gigantic and accumulated in dams with different degrees of toxicity can be transformed into major tragedies. In the state of Minas Gerais, serious accidents with dams have been frequently repeated: 2001, 2003, 2007, 2008, 2014, with several deaths and environmental destruction [8]. This study aimed to investigate the effects of dam bursting on the production of outpatient health services and on the expenses incurred from 2013 to 2017 in Mariana and 2017 to 2021 in Brumadinho.

## Methods

This is a cross-sectional, quantitative study on the production and expenditure of ambulatory services in the municipalities of Mariana and Brumadinho.

The study of production and spending on ambulatory care in the two municipalities affected by the mining disaster makes it possible to identify situations of imbalance to support planning, management, and evaluation processes for the care provided to the population. Ambulatory care is the type of service provided by health personnel to clients in outpatient clinics on a non-hospitalized basis, according to the basic health terminology of the Ministry of Health, Brazil.

Thus, to characterize the production and expenditure of ambulatory services, the variables were used:

- Approved Quantity: Quantity of procedures approved for payment by the Health Departments.
- Approved Value: Approved value for payment by the Health Secretariats.

Data on the production and expenses of outpatient services in Mariana and Brumadinho were obtained from the Ambulatory Information System (AIS) available at the Computer Department of SUS (http://datasus.saude.gov.br).

To evaluate the impact of events on the outcomes of ambulatory care, data were collected monthly, organized in Microsoft Office Excel spreadsheets, and expressed in time series charts listing the years 2013, 2014, 2015, 2016, 2017 regarding Mariana and 2017, 2018, 2019, 2020, and 2021 regarding Brumadinho. The months of November 2015 and January 2019 were the base months of comparison, i.e., month 0, since these were the months when the Mariana and Brumadinho disasters occurred. The previous months were identified from 24 to -1 and the subsequent months from + 1 to + 24.

Segmented regression analysis was performed to determine disturbances and variations during the study period. Segmented regression is a method of analysis in which the independent variable is partitioned into intervals on a separate line segment and adjusted for each interval. It is used when the independent variables, gathered in different groups, exhibit relationships between the variables in these regions.

The formula for the regression line coefficients used to calculate the variables was as follows:

Variable analyzed = constant + reference + disaster + month after disaster.

$${\bf{Y}} = {\bf{\beta 0}} + {\bf{\beta 1X1}} + {\bf{\beta 2X2}} + {\bf{\beta 3X3}}$$

The first straight line, which is situated in the reference months from − 24 to -1, is expressed by β1 and the gradient expressed by β2, year of the disaster. The reference months + 1 through + 24 are the post-disaster months, which is β3. Thus, when we mention betas (β) we will refer to pre-disaster, disaster, and post-disaster.

The segmented regression analysis enabled us to assess the data’s sensitivity and understand how the disasters may have affected production and outpatient spending in the municipalities of Mariana and Brumadinho. This analysis was crucial for identifying the behavior of the study variables before and after the mining disaster and for determining whether the mining disaster impacted ambulatory production.

Data were also calculated using absolute numbers, which generated relative numbers that could provide a better idea of the data in terms of comparison and confirmation of the regression calculations. The display of absolute numbers can provide foundations and elements so that those interested have a greater set of subsidies and resources for understanding the phenomenon of the Mariana and Brumadinho disasters. The statistics described were used as a complement to show the figures for ambulatory production and the amounts paid by the Unified Health System (UHS) in the municipalities of Mariana and Brumadinho.

According to the recommendations of the Resolution of the National Health Council (NHC) No. 466, December 12, 2012, the ethical principles of research involving human beings were respected, and the approval of the research ethics committee was waived because the study was conducted from secondary data, of public access, without the possibility of individual identification of the information. The study had, in turn, a Statement of Responsibility signed by those responsible for the research.

## Results

**Fig. 1:**
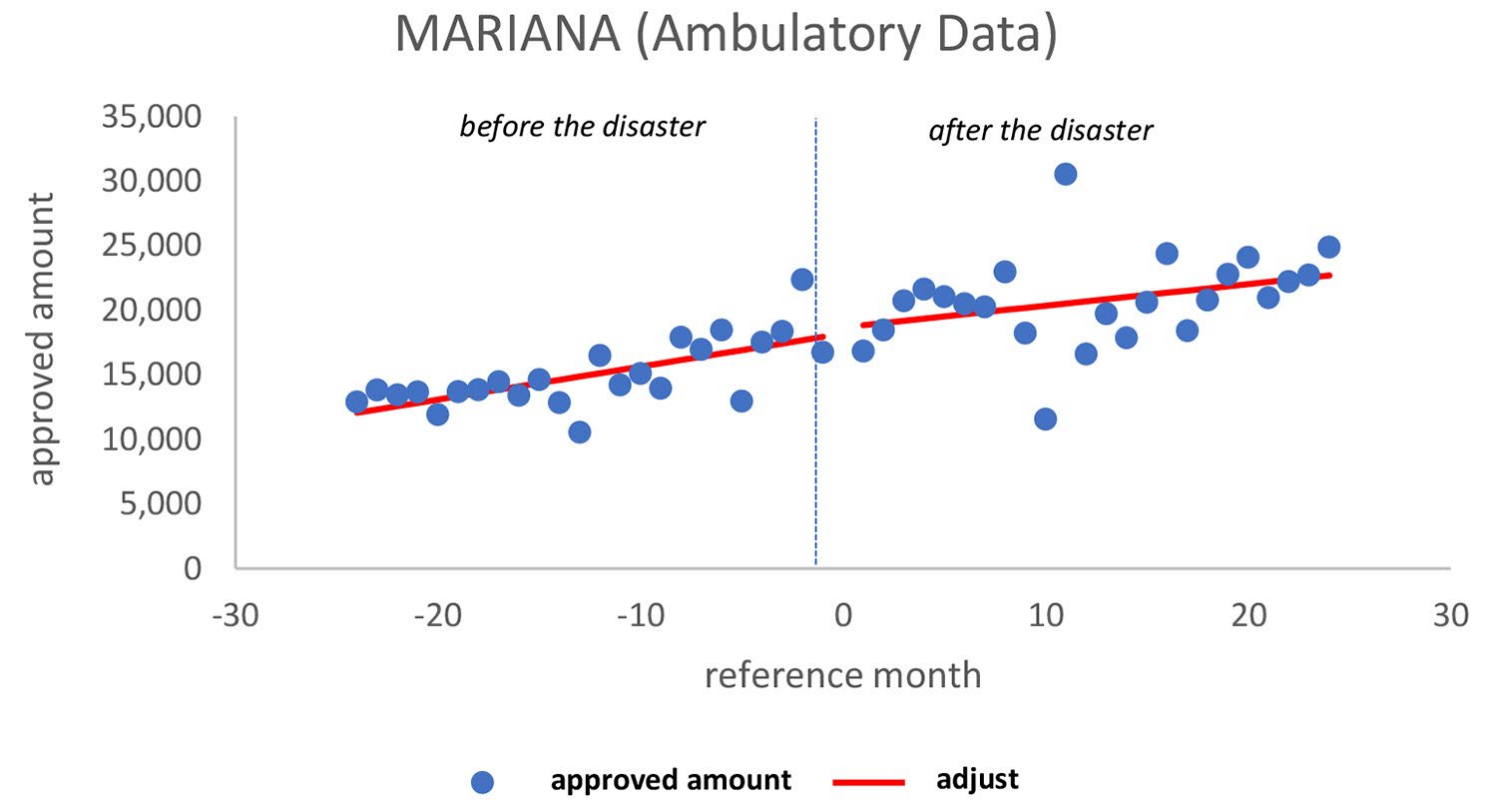
Ambulatory approved amount in Mariana Source: Developed by the authors

In Mariana, there was a tendency to increase the approved amount of ambulatory production in the period before the disaster (*p* = 0.004), no significant change soon after (*p* = 0.769) and no significant change in the pattern in the later period (*p* = 0.457) (Fig. 1). There is no breakpoint that could justify a change, suggesting a continuation of the upward trend that began before the disaster.

**Fig. 2:**
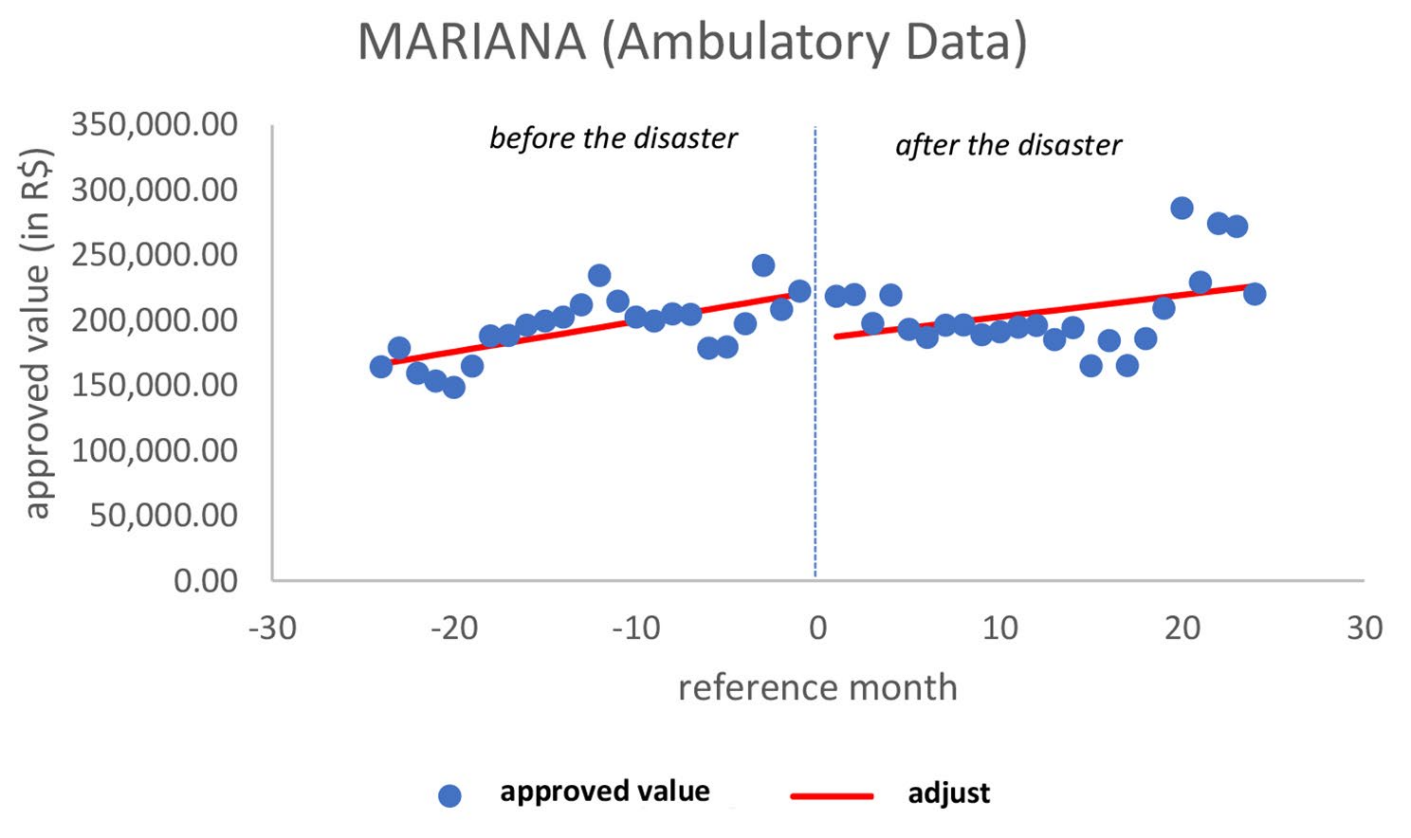
Ambulatory approved value in Mariana Source: Developed by the authors

When the approved values were analyzed, there was a tendency to increase the value in the period before the disaster (*p* = 0.003), a significant decrease in the approved value immediately after (*p* = 0.017) but no significant change in the pattern in the later period (*p* = 0.539). In this figure, a significant breakpoint indicates a consistent change on the disaster day (Fig. 2).

**Fig. 3:**
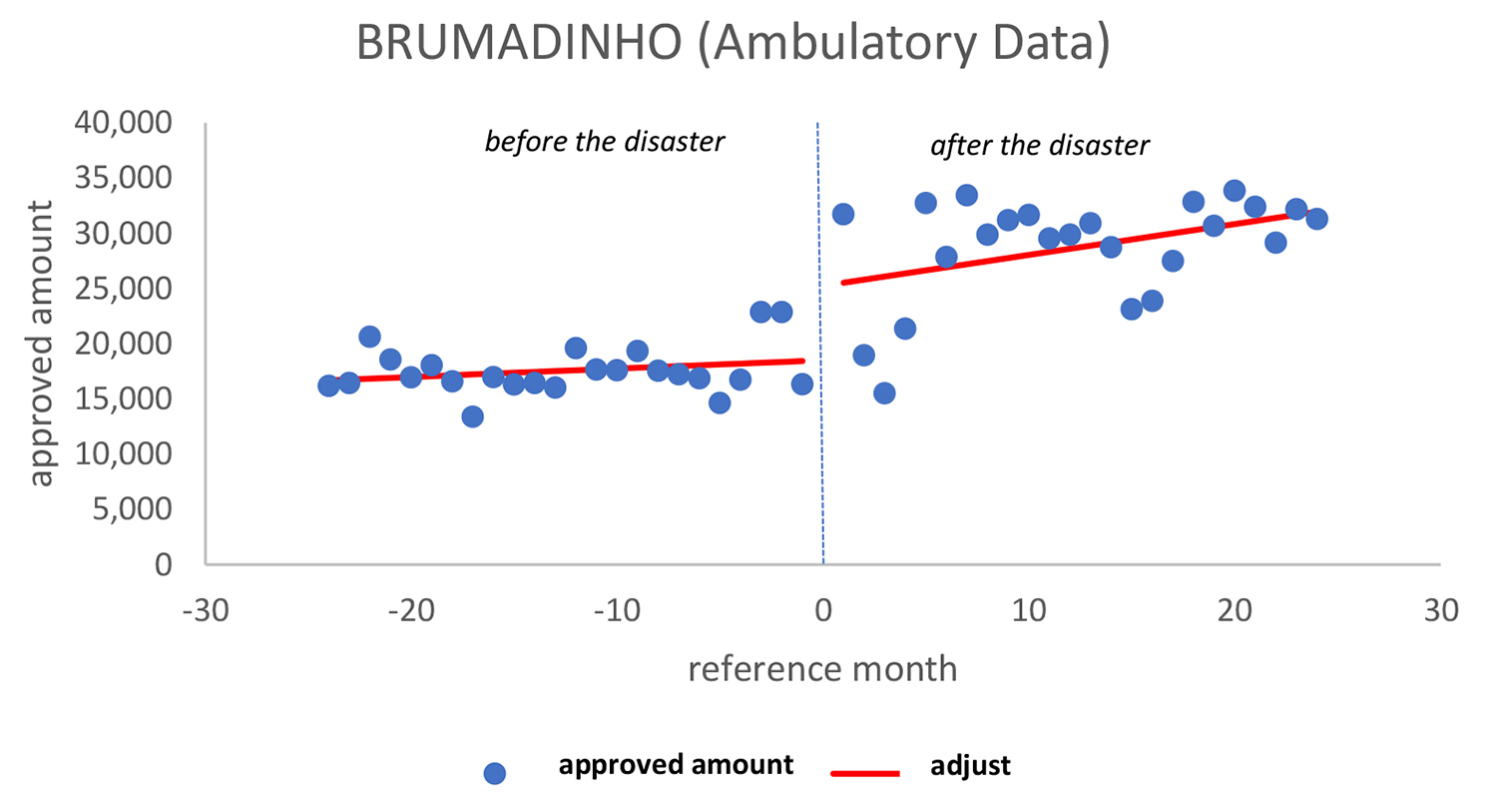
Ambulatory approved amount in Brumadinho Source: Developed by the authors

In the case of Brumadinho, when analyzing the approved amount, we observed constant attendance in the previous period (*p* = 0.470), a significant increase soon after (*p* = 0.003), as there was a breakpoint between disasters, which continued to increase in the later period (*p* = 0.172), demonstrating that the subsequent 24 months was a continuation of the ascendency of the day of the disaster (Fig. 3).

**Fig. 4:**
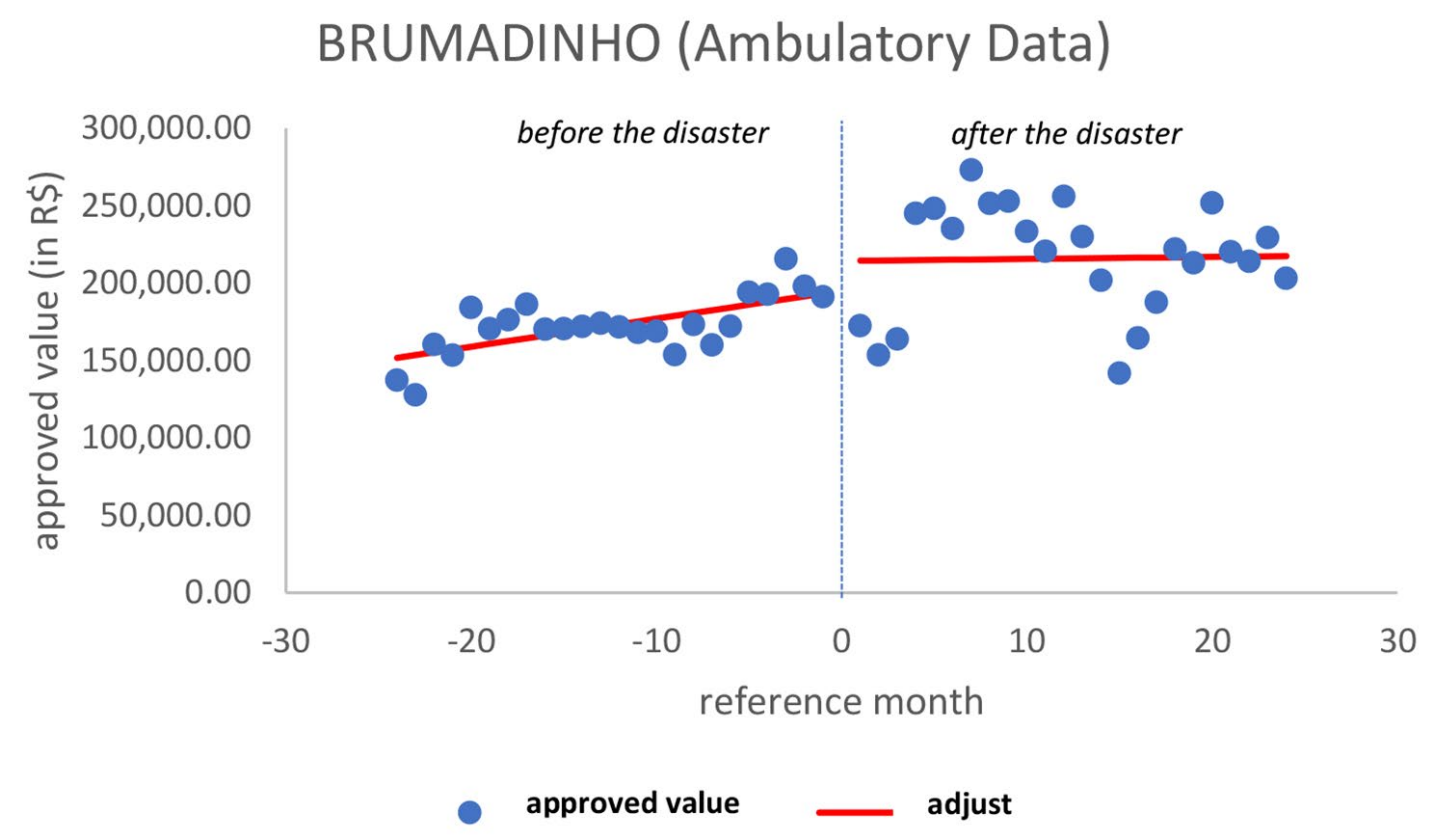
Ambulatory approved value in Brumadinho Source: Developed by the authors

When analyzing the approved values, there was an increasing trend in the previous period (*p* = 0.033), soon after there was no significant change (*p* = 0.254) and no significant change in the pattern in the later period (*p* = 0.155), although apparently there is a breakpoint on the day of the disaster, which is not significant (Fig. 4).

The data in Table 1 shows an increase of 31.37% in Mariana and 3.36% in Brumadinho, which was actually lower when compared to the percentage population change in the year for Mariana (2013–2107) and lower in the year for Brumadinho (2017–2020).

**Table 1:** UHS ambulatory production of the Approved Value, total population, Mariana and Brumadinho

	Before	After	Percentage variation
Mariana	7.105.617,03	9.334.581,77	31.37%
Brumadinho	5.082.894,59	5.253.925,59	3.36%
Minas Gerais 2013 − 2017	3.396.745.992,16	3.533.177.664,60	4.02%
Minas Gerais 2017–2020	3.719.706.736,14	3.765.216.768,08	1.22%

Source: DATASUS-Information Technology at the Service of the UHS

Table 2 shows the ambulatory care data for Mariana and Brumadinho in relation to the population of Minas Gerais during the study period. The results show that there was a 1.12% increase in the number of treatments in Mariana in the post-disaster period, whereas the population of Minas Gerais fell by -2.97% in the number of treatments. The population of Brumadinho saw a drop in the number of people assisted, but when compared to the population of Minas Gerais in the years before and after the disaster, it was much smaller: in the disaster’s city (Brumadinho), the drop was − 2.33% and in Minas Gerais it was − 14.65%.

**Table 2:** UHS ambulatory production by outpatient care, Minas Gerais, Mariana and Brumadinho

	After	Before	Percentage variation
Mariana	1.259.121	1.273.272	1.12%
Brumadinho	1.225.299	1.196.769	-2.33%
Minas Gerais 2015 − 2017	888.078.226	861.739.279	-2.97%
Minas Gerais 2017–2020	757.113.312	646.214.613	-14.65%

Source: DATASUS-Information Technology at the Service of the UHS

Table 3 shows the rate of health spending according to population estimates provided by the Brazilian Institute of Geography and Statistics (IBGE). A glance at the data reveals that, before the disaster in 2014 and 2015, spending was lower in Mariana. The disaster happened on November 5, 2015, and spending grew from 2016 onward, and has remained so.

**Table 3:** Health expenditure according to IBGE population estimates for Mariana, in Real/Brazil

	2014	2015	2016	2017
Mariana	R$ 56,94	R$ 65,66	R$ 76,89	R$ 81,11

Source: Elaborated by the authors

Table 4 shows health expenditures according to ​​​​​​​the IBGE population estimates for Brumadinho. Looking at the data before the Brumadinho disaster, which occurred on January 25, 2019, the results show that in the disaster year, there was a significant increase in the number of per capita expenses, which fell in the following year.

**Table 4:** Health expenditure according to IBGE population estimates for Brumadinho, in Real/Brazil

	2017	2018	2019	2020
Brumadinho	R$ 66,27	R$ 63,45	R$ 71,98	R$ 58,22

Source: Elaborated by the authors

Table 5 shows health expenditures before and after the disaster in Mariana and Brumadinho, approximately two years before and two years after the disaster, divided by the population estimate for the period, giving us a brief perception of an increase in spending in both Mariana and Brumadinho in the aftermath of the disaster. While in Mariana there was a substantial increase of R$35.11 per person, in Brumadinho there was an increase of R$0.58 per person.

**Table 5:** Health expenditure before and after the disaster in Mariana and Brumadinho, according to population estimates

	Before the disaster	After the disaster
Mariana	R$ 120,84	R$ 155,95
Brumadinho	R$ 128,62	R$ 129,20

Source: Elaborated by the authors

The data in Table 6 ​​​​​​​show ambulatory care in the municipalities of Mariana and Brumadinho, as well as in the state of Minas Gerais, obtained from the National Supplementary Health Agency via DATASUS. According to the data obtained, there was a -41.57% drop in the percentage of outpatient access to private healthcare after the disaster in the city of Mariana, which was higher than the drop in the state of Minas Gerais over the same period (2013–2017), which was − 35.85%. In contrast, Brumadinho saw an increase of 31.94% in outpatient service access, unlike Minas Gerais, which saw a drop of -4.76%.

**Table 6:** Ambulatory medical care/supplementary health by municipality Mariana and Brumadinho, and state Minas Gerais

	Before the disaster	After the disaster	Percentage variation
Mariana	356	208	-41.57%
Brumadinho	382	504	31.94%
Minas Gerais 2013–2017	3.902.901	2.503.631	-35.85%
Minas Gerais 2017–2020	3.188.325	3.036.484	-4.76%

Source: Elaborated by the authors

## Discussion

When we analyzed the approved quantities, Mariana showed no significant change immediately after the disaster and no significant change in the pattern in the later period, whereas in Brumadinho, there was a significant increase immediately after the disaster, which continued to grow, but no significant change in the pattern in the subsequent period in relation to the month of the disaster, which in turn implies a change in the entire period.

In the case of the approved values only in Mariana in the month of the disaster, a significant decrease is observed. These values in the later period are similar to those in the previous period, but without a significant growth trend, which indicates that the day of the disaster was crucial in the arrangement of the subsequent values, implying a continuity of the line that grows without significance.

In Brumadinho, there was an increase in the approved quantity in the month of the disaster that continued in the subsequent period.

Comparing this with population data from DATASUS, the results show that spending before the disaster for the total population of Minas Gerais was lower than spending after the disaster, when compared to Mariana and Brumadinho, which also saw an increase in spending in the post-disaster period. It can be seen that the figures for both cities were higher than the total population of Minas Gerais, almost three times the percentage with Brumadinho, at 3.36%, compared to 1.22% in the population years of Minas Gerais in the period from 2017 to 2020. From 2013 to 2017, spending on ambulatory care in Minas Gerais increased by 4.22% and in Mariana by 31.37%. The comparison with state demographic data corroborates that the absence of statistical significance, i.e., the line, did not change and maintained an upward trend, indicating the impact that Mariana and Brumadinho had on ambulatory care provided by the Unified Health System. In practical terms, the lack of significance does not imply that there was no impact from the disaster, since we are working with trends. Thus, the trend data maintain a direction that can apparently be translated and show a possible effect, which even if it is not significant may have some directional effect on the impact, since the growth trend persisted from before the disaster with Brumadinho. These findings corroborate those described in the literature where disasters increase spending, which starts to compete and interfere with other social sectors [9]. Researchers attribute increases in spending less aggressively because of the lack of transfers from the Ministry of Health and the state government to affected locations [10].

The data on ambulatory care, in absolute and relative terms, is significant when compared with the population of Minas Gerais in the years corresponding to the disaster in each city. While in Mariana there was an increase of 1.12%, in the state of Minas Gerais there was a drop of -2.97%, which shows that the disaster had consequences for the city’s ambulatory service. With Brumadinho, the occurrence of the disaster led to a drop in care, but when compared with the population data, the drop was significantly less, so in the disaster’s city there was a drop of -2.33%, and in the state of Minas Gerais the drop was − 14.65%. Besides immediate damage, disasters bring with them important changes in morbidity and mortality, increasing the rate of illness and consequently increasing the need for a range of health services, which has repercussions such as higher rates of medical consultations [11, 12].

The analysis of the expenditure resulting from the Mariana and Brumadinho disasters shows that there was a change in spending in absolute and percentage terms. In the month of the Mariana disaster, we have a breakpoint that shows that there was a drop in spending, but then it picks up again, so apparently the expenditure figures are significant, and in absolute and percentage terms in the post-disaster period these figures are suggestive. In Brumadinho, the percentage figures show a slight variation in spending in the post-disaster period in percentage and absolute terms, while the segmented regression line is more generous and points to an upward trend in the pre-disaster period and an increase in the disaster period, which continues throughout the period. Research into disasters and economic and spending analyses show an increase in spending in the post-disaster period. These analyses show what had already been announced frequently in various media, but which had not been clarified in numerical terms [9]. Studies of the population over 65 years of age show that exposure to too many natural disasters can have an important effect on the utilization and cost of health care in the United States, and that such occurrences imply substantial spending on health care for this important population niche [13]. The data from the survey corroborates this information and provides a basis for its confirmation.

The supplementary health data from Mariana and Brumadinho allow for some inferences and quick conjectures about their role in the disaster, both in absolute and percentage terms, so it is possible to say that their role was insignificant, with Mariana showing a drop in the number of patients treated by ambulatory medical care. Thus, with a 41.57% drop in private healthcare in Mariana and a 1.12% increase in the public healthcare network, although the segmented regression data points to a maintenance of the standard of care in the public network, this does not explain why there was a continuation of expenses that came from before the disaster and which may have been maintained by the disaster. In the case of Brumadinho, the supplementary health service saw an increase of 31.94% and a percentage drop of 2.33% in the public service, which could mean a transfer of care to the private health service. However, when we look at the segmented regression line for Brumadinho, we see that the number of attendances has a significant upward trend in the post-disaster period, which credits the UHS with a growing increase in the number of attendances. In any case, an increase in the private service is not ruled out, but a detailed study will not be performed because it is beyond the scope of this work.

Data from the Getúlio Vargas Foundation show that the municipalities affected by the Samarco mining disaster presented an abrupt change in the expected pattern of health and in the cycles of vectors and disease hosts months after the event [14]. These same data, when related to the affected communities, showed that the incidence of diseases and illnesses per 100,000 inhabitants in the 45 municipalities considered affected and 85 controls, between 2015 and 2019, projected a three-year decrease in the life expectancy of the exposed population compared with the control population. Thus, multiple diseases and risk factors to which the population was exposed are determining factors in the expected pattern of morbidity and mortality, which will result in changing the pattern of illnesses and which converge with the data that was obtained in the research developed, both with regard to the pattern of spending and the pattern of care.

In Puerto Rico, disasters such as Hurricane Maria have brought intense devastation that is reflected in the increase in deaths of people in general and mainly low-income people, as expressed in the available municipal data series [15]. In addition, there is information that estimates higher morbidity in health services, which in theory are related to the effects of the disaster that generate a deep tear in the core of public health. These effects are felt in the extension of mental health, which can be reflected in post-traumatic stress disorder, anxiety, and suicidal and depressive ideation [16].

These findings are corroborated by information published in the epidemiological bulletin of the Secretariat of Health Surveillance of the Ministry of Health of Brazil, which states that in the disaster, a series of alterations in the pattern of care arise and diversify under the premise of diseases that affect the population in the short, medium, and long term. As evidenced by the bulletin, our study also showed that in Brumadinho, the approved amount of ambulatory care increased after the mining disaster [17]. Of these short-term care, most are due to the rupture of the dam that resulted in immediate deaths and casualties. The medium- and long-term care is due to direct and indirect exogenous contamination by the material present in the grafted tailings, mainly of metallic nature, and that may still incur various diseases in this same population. In addition to the impact on mental health due to the number of lives lost and the sudden change in the population’s daily lives [17].

The damage of disasters that manifest themselves in the short term, range from hours to days, and include besides the injured, the dead and light and serious injuries. In ambulatory care, we have short- and long-term impacts that are conceived after the accident and that have an immediate, non-emergency character. Such care involves water-borne diseases and those transmitted by vectors, such as dengue and malaria. Long-term impacts include emotional and psychosocial processes, mostly experienced through social abandonment in the reconstruction and recovery of the affected areas [18, 19]. Disasters such as the one that struck Brumadinho can extend for miles and cause various damages to the surroundings and the population, resulting in various impacts on the ecosystem, thus producing conditions for the transmission of infectious and contagious diseases present in the region. In addition, the disaster may aggravate pre-existing chronic diseases in the population, such as diabetes, hypertension, cardiovascular diseases, and renal failure [20].

In addition, towns in the surrounding area have suffered and are still suffering from the consequences of mining disasters, such as Barra Longa, where 250 people were directly injured and 55 ill, and where approximately 7,727 consultations were conducted between 2015 and 2018. In the disaster year, in Barra Longa, 15.2% of the reasons for consultations were upper airway infections (URTI), parasitosis, hypertension, and dermatitis, followed by 8.9%, 8.6%, and 1.5%, respectively. In the following year (2016), the same diseases had the same placements with a variation in the percentage of predominance of the population, so URTI had 21.2% of the reasons for attendance, parasitosis 9.5%, hypertension 4.2%, and dermatitis 1.9%. Research data shows that exposure to dust after the collapse has led to consequences such as URTI, allergies, and depression, as well as worsening of diseases such as URTI, dermatitis/dermatosis, and allergies. URTIs, allergies, and depression were the most cited as worsening conditions over the last 2.5 years in Barra Longa, which may be related to the 2015 disaster. The data also warn that psychosocial and behavioral disorders, cardiovascular diseases, and malnutrition have somewhat worsened. In addition, the data obtained in 2016 nine months after the disaster are similar to those obtained 2.5 months after the dam burst [21].

According to the surveys, residents in 45 municipalities were each deprived of an average of 2.39 years of life because of disability, considering a set of 75 diseases with accumulated incidences [22]. Some of the population exposed to the disaster has a low levels of education, low income, underemployment, and poor sanitation conditions. Contact with the effects of the disaster, as well as the chemical substances harmful to health caused by the disaster, is an additional risk factor that has exacerbated the vulnerability of those affected. In addition, according to the data obtained, respiratory diseases, neoplasms, and violence account for 92% of all identified problems [22].

Although the Mariana and Brumadinho disasters share similarities, they also have idiosyncrasies that make them different. Although Mariana was a disaster of great magnitude and its environmental effects were of great intensity, the human impact felt in the short term was less. While Brumadinho had a death toll of over 300 victims, Mariana had a death toll of 19, which is much lower in quantitative terms. Both the municipalities of Mariana and Brumadinho had their surrounding towns affected by the dam collapse, which in practice meant that the Doce (Mariana) and Paraopeba (Brumadinho) rivers were hubs for the spread of diseases and illnesses that appeared in the towns near the disaster site. In this sense, the Paraopeba River in Brumadinho, which is approximately 250 km long, was a focus of attention for the spread of contamination along its course, which has about 147 and 424 communities (indigenous, quilombolas, foresters, and artisanal fishers), and involved surveillance of water quality in the 18 municipalities crossed by it. Mariana reached 36 municipalities with Samarco’s tailings sludge along a 663 km stretch to the mouth of the Doce River. In a municipality close to Mariana, Barra Longa, they revealed a series of existing diseases and the appearance of new ones in a scenario of overlapping threats, diseases, and damage [23].

Our findings show that care in Mariana increased in absolute and relative terms, which according to the segmented regression line had already been happening, i.e., there was already a maintenance of the growth pattern, which may be a coincidence or maybe because of the disaster; however, according to data in the literature, it can be conjectured that this increase was real [12]. With Brumadinho, the data show an increase in the number of consultations, which is significant from the point of view of the segmented regression line and is not shown by the absolute and relative data. However, because there has been a decrease in the number of consultations in the state, this value is higher than its drop. In addition, in this case, previous research confirmed an increase in the number of medical consultations [24]. As a city hit by the disaster, Brumadinho had the prerogative of having a framework of integrated preventive actions that could somewhat mitigate the impact on the city. These public actions, under the SUS, included health surveillance mechanisms (focusing on chemical contamination and mental health, linking epidemiological and health surveillance with basic care, Emergency Care Unit - ECU - and hospital) and health care (ECU, hospital, laboratories, Psychosocial Care Centers - PSCC, Integrative and Complementary Health Practices Centers - ICHPC - basic health units - BHU Family Health Strategy - FHS, and Family Health Support Centers - FHSC), which had the support of the Ministry of Health and the Minas Gerais Health Department [12]. All this equipment was important for building a set of immediate, integrated, and incorporated SUS actions that expanded surveillance and health care activities in the aftermath of the disaster, which helped with the adversities that arose after the event.

Many studies point to a higher incidence of chronic diseases after disasters, and these values are an indication that the continued growth in the amount approved and in the values approved in Mariana and Brumadinho are not a mere reflection of what was occurring, but an expression of the impact of the disaster on ambulatory care in the Unified Health System. It is not known for sure what motivated the growth before, but the continuity was possibly due to the disaster; for example, earthquakes bring with them a higher incidence in the mortality rate as myocardial infarction and stroke in the first month of the accident and up to three years in high-income countries [25]. Recent studies have indicated increased rates of psychiatric disorders following natural disasters [26]. They show that in Brumadinho, there is a high multi-morbidity in the studied population, which is influenced by socio-demographic factors and area of residence, which demands adequate long-term management of multiple chronic diseases by health professionals because it constitutes a challenge for patients, professionals, and society as a whole [27]. These data corroborate that the upward trend of the approved amount on the day of the disaster in Brumadinho and its non-change soon after are motivated by factors from the day of the disaster. Similarly, approved values that started high before the disaster remained so because such an event occurred.

Regarding Mariana and infectious diseases, whether transmitted by vectors, such as dengue, hepatitis A, and diarrhea, it was notable that the number of dengue cases has increased, especially in the city of Barra Longa, where the number of dengue cases had a sudden increase. In a survey conducted, about 6.6% of respondents said they had dengue, approximately 31 inhabitants, and after the survey, the city had an outbreak of the disease, which was reported by officials from the Barra Longa Municipal Health Department. Dengue fever occurred only in urban areas. There was one case of Zika virus, a resident of the center, and one case of Schistosomiasis, Chagas, or Leishmaniasis. There were no cases of Hepatitis A, Chikungunya or Leptospirosis [28]. It was noticeable that there was a latent concern about the cleanliness of the affected places, especially the districts of Bento Rodrigues and Paracatu de Baixo in Mariana, the district of Gesteira, and the seat of Barra Longa. These locations could become breeding grounds for disease vectors such as Dengue, Chikungunya, Zika Virus, Schistosomiasis, Chagas, Leishmaniasis and problems with venomous animals [28].

Thus, a correlation exists between Mariana and Brumadinho in terms of the amount approved for ambulatory care and the post-disaster period, which culminates in an increase in the number of treatments. Positive correlation between Mariana and Brumadinho in the pre-disaster period concerning the amount approved for both. And that ends up suffering a decrease on the day of the disaster, in the case of Mariana, and an increase in Brumadinho, remaining the same throughout the period after the disaster, finding similar reflections in the scientific literature, and that are fundamental in the organization, preparation, and readiness to deal with disasters and adverse events [29].

## Conclusions

In Mariana, the approved ambulatory care amounts maintained a growth trend throughout the period, suggesting that they were not affected by the disaster. In contrast, Brumadinho showed a significant increase in these amounts soon after, which continued to grow in the subsequent period, suggesting a change in the use of ambulatory services due to the disaster.

In Mariana, the approved values showed a significant reduction right after the disaster, which continued in the subsequent period. In Brumadinho, the increasing trend was maintained in all three periods without changing the pattern.

Corroborating the information from the segmented regression lines, which sometimes showed possible significance, the absolute and percentage data from the cities of Mariana and Brumadinho suggest an effect of the disaster on the hospitalizations and health expenses suffered by the population. The city of Mariana, for example, showed data that possibly shows a change in gross data, but which in statistical terms (segmented regression line) suggests a continuation of previous spending. In the city of Brumadinho, despite the percentage and absolute data being less expressive, the segmented regression line suggests a change in hospitalization and expenditure.

Based on the findings, it was possible to understand that although disasters exert an influence that may have some effect on the health system, the lack of significance sometimes cannot be interpreted as a lack of impact on the disaster. The segmented regression line outlines some effects that are not conclusive but indicative of a numerical interpretation and a trend interpretation.

The information is suggestive and requires new data to be incorporated so that the research can recommend more decisive statements. In addition, the data should be monitored more closely to obtain a more conclusive view.

Limitations of the study suggest the lack of specification of outpatient care in terms of illnesses, which could lead to a better analysis of the profile of care and which, for technical reasons, was an impediment to this analysis.

## Data Availability

Availability of data sets used and/or analyzed during the current study is available from the corresponding author upon reasonable request.

## Abbreviations

AVABRUM: Association of Families of Victims and People Affected by the Córrego do Feijão Dam
NHC: National Health Council
AIS: Ambulatory Information System
UHS: Unified Health System
IBGE: Brazilian Institute of Geography and Statistics
URTI: Upper Respiratory Tract Infections
ECU: Emergency Care Unit
PSCC: Psychosocial Care Centers
ICHPC: Integrative and Complementary Health Practices Centers.
BHU: Basic Health Units
FHS: Family Health Strategy
FHSC: Family Health Support Centers
